# A qualitative study of children’s snack food packaging perceptions and preferences

**DOI:** 10.1186/1471-2458-14-1274

**Published:** 2014-12-15

**Authors:** Paola Letona, Violeta Chacon, Christina Roberto, Joaquin Barnoya

**Affiliations:** Cardiovascular Unit of Guatemala, 5a. Avenida 6-22 zona 11, Guatemala City, Guatemala; INCAP Research Center for the Prevention of Chronic Diseases, Institute of Nutrition of Central America and Panama, Calzada Roosevelt 6-25 zona 11, Guatemala City, Guatemala; Department of Social & Behavioral Sciences, Harvard School of Public Health, 9 Bow Street, Cambridge, MA 02138 USA; Division of Public Health Sciences, Department of Surgery, Washington University in St. Louis, 660 S. Euclid Ave, Campus Box 6100, St Louis, MO 63110 USA

**Keywords:** Pediatric obesity, Low/middle income countries, Marketing, Packaging

## Abstract

**Background:**

Food marketing is pervasive in high- and low/middle-income countries and is recognized as a significant risk factor for childhood obesity. Although food packaging is one of the most important marketing tools to persuade consumers at the point-of-sale, scant research has examined how it influences children’s perceptions. This study was conducted in Guatemala and aimed to understand which snack foods are the most frequently purchased by children and how aspects of food packaging influence their product perceptions.

**Methods:**

Six activity-based focus groups were conducted in two elementary public schools with thirty-seven children (Grades 1 through 6, age range 7–12 years old). During each focus group, children participated in three activities: 1) list their most frequently purchased food products; 2) select the picture of their favorite product, the packaging they liked best, and the product they thought was the healthiest from eight choices; and 3) draw the package of a new snack.

**Results:**

Children reported purchasing salty snacks most frequently. Most children chose their favorite product based on taste perceptions, which can be influenced by food packaging. Visual elements influenced children’s selection of favorite packaging (i.e., characters, colors) and healthiest product (i.e., images), and persuaded some children to incorrectly think certain foods contained healthy ingredients. When children generated their own drawings of a new product, the most frequently included packaging elements in the drawings were product name, price, product image and characters, suggesting those aspects of the food packaging were most significant to them.

**Conclusions:**

Policies regulating package content and design are required to discourage consumption of unhealthy snacks. This might be another public health strategy that can aid to halt the obesity epidemic.

## Background

Food marketing is now recognized as a risk factor for child obesity and noncommunicable diseases (NCDs) [1]. Marketing of high-energy, low nutrient foods is pervasive in high- and low/middle-income countries (LMICs) and it has been shown to influence children’s food preferences, purchase requests and consumption [2–5].

Television, Internet and digital child-oriented food marketing has been previously documented in different countries [6–8]. However, scant research has focused on food packaging, another marketing strategy used at the point-of-sale (POS) [9]. The package is one of the most important factors persuading consumers at the POS [10] and has become a significant component of branding, positioning and communication [11]. It also attracts consumer’s attention, enhances product image, influences consumer’s perception about the product, provides information, distinguishes one product from another, and stimulates impulsive buying behavior [12, 13].

Packaging elements can be divided into visual (e.g., graphic, color, shape, size) and informational elements (e.g., product name, brand, producer/country, product information, special offers) [14]. Food marketers have proven successful when targeting children through visual elements. Research has found that licensed characters [15–17], branding [18], decorative designs [19], and sports celebrity endorsements [20] on packaging influence children’s taste and food preferences. In addition, informational elements such as health claims, lead children to prefer the taste of products with such claims and to perceive them as healthier than those without claims [20–22].

Given the lack of evidence on the influence of on-package food marketing techniques in LMICs, we aimed to understand how Guatemalan children from public schools (attended by children of lower socioeconomic status (SES) relative to private schools), perceive food packaging. We were interested in studying those of lower SES because they might have lower media literacy and therefore be more susceptible to the persuasive intent of marketing [3]. This study sought to answer five research questions: 1) What are the most frequently purchased food products by children? 2) What leads children to select their favorite product? 3) What leads children to select their favorite packaging? 4) How do children decide if a product is healthy? and 5) What packaging elements are most significant to children when drawing the packaging of a new snack?

## Methods

We used a parallel mixed methods approach of activity-based focus groups. We included three activities (i.e., list, picture selection, drawing) to make focus groups more fun, maintain children’s attention, give them time to think about their responses, and enhance discussion [23–25]. This enabled us to record each child’s selections for quantitative and qualitative analysis. The study protocol was approved by the Institutional Ethics Committee of the Institute of Nutrition of Central America and Panama (INCAP) in Guatemala.

### Participants

The participants were selected using convenience sampling of two low-income elementary public schools in Mixco, an urban community in the Department of Guatemala where 68% of schoolchildren attend public schools [26]. Previous publications in Guatemala have used type of school (public or private) to classify children in public schools as being of low SES [27, 28]. Public schools in Mixco have similar characteristics across the schools; therefore children selected were not likely to be different to children from other public schools in the area. Permission was obtained from the School District Supervisor to conduct activity-based focus groups at the schools. At each school, the principal provided the lists of students enrolled in elementary school (Grades 1 through 6, age range 7–12 years old). The lists were then stratified by gender to guarantee that the same number of girls and boys were selected using a random digit generator. Parents of the selected children were invited to a school meeting where the study, procedures, and consent form were explained. Only children with written parental consent and child verbal assent were recruited.

### Procedure

A total of 6 activity-based focus groups were conducted in September 2012, three in each school: one with first and second graders (7–8 years old), one with third and fourth graders (9–10 years old), and one with fifth and sixth graders (11–12 years old). Each focus group consisted of 4 to 9 participants and lasted approximately 60 to 90 minutes. All focus groups were conducted privately in an empty classroom and facilitated by a moderator with the help of a research assistant who provided technical support. Participants received a snack after the focus group as a compensation for their time.

### Discussion guide

All focus groups were conducted using the same customized discussion guide based on the study’s objectives. After a short introduction, participants discussed their favorite television shows as a warm up, prior to participating in the three main activities. In the first activity, each child received a pencil and a sheet of paper and was asked to write down the name of the foods and beverages they most frequently buy in stores inside and outside the school. We did not specify the time of day (e.g., before, during or after school) or request the children to include products they ask their parents to buy them because it is common for parents to provide children with money to buy food on their own at stores [29]. In the second activity, each child was given a set of eight colored photos of different types of snacks and beverages that most children have tried, but are not exposed to them on a daily basis because they are not usually available in the school food kiosk: salty packaged snacks (i.e., Cheetos, Lay’s Potato Chips, Fiesta Snax), cookies (i.e., Cremas, Lors), fruit drinks (i.e., Chupi Frut, V8 Splash), and a regular soda (i.e., Grapette Soda). Five of those products had child-targeted marketing techniques on the packaging (i.e., promotional characters, premium offers, movie tie-ins), the other three did not (Table 1). Pictures of products that were very familiar to children were excluded to reduce potential bias due to prior experience with the product [16]. Children were only shown pictures of the front of the package; no nutritional information about the products was provided. Children were asked to observe all photos carefully and select their favorite product, favorite packaging and what they considered to be the healthiest product. Children were then asked to explain why they chose it. In the third activity, each child received two sheets of paper, one pencil, and a box of crayons. Children were told a story that a food company was going to launch a new cheese-flavored packaged snack and that they wanted their opinion about how to design the packaging so that children their same age would buy it. Children were then asked to draw the packaging (front and back) for the new snack and to explain their drawing. At the end, all the drawings were collected. Research assistants then reviewed each drawing and recorded the packaging elements (i.e., visual, informational) drawn.

**Table 1:** **Children**’**s selection of favorite product and packaging, and healthiest product**

Product name	Child-targeted marketing strategies	Favorite product	Favorite packaging	Healthiest product
		n (%)	n (%)	n (%)
Cheetos	Promotional character	9 (24.4)	4 (10.8)	2 (5.4)
Lay’s Potato Chips	Promotional character, premium offer, movie tie-in	6 (16.2)	7 (18.9)	0 (0.0)
Fiesta Snax	None	7 (18.9)	4 (10.8)	0 (0.0)
Lors	None	2 (5.4)	4 (10.8)	2 (5.4)
Cremas	Promotional character, premium offer, movie tie-in	1 (2.7)	0 (0.0)	2 (5.4)
Chupi Frut	Promotional character, movie tie-in	3 (8.1)	5 (13.6)	8 (21.6)
V8 Splash	None	3 (8.1)	7 (18.9)	20 (54.1)
Grapette Soda	Promotional character, movie tie-in	6 (16.2)	6 (16.2)	3 (8.1)

### Data collection and analysis

Descriptive statistics were used to summarize the type of products children reported purchasing most frequently, favorite products and packaging from the samples provided, products perceived as the healthiest, and package elements included in the drawings. STATA software was used for data analysis.

Focus groups were audio-recorded and transcribed verbatim in Spanish. The transcripts were closely reviewed after data collection and a coding scheme was developed based on themes that emerged that addressed the research questions. The transcripts were organized, coded, and analyzed using ATLAS.ti (version 6.2). Analyses were conducted in Spanish by a bilingual member of our team, who also translated excerpts selected for quotations into English.

## Results

Thirty-seven children participated in the focus groups, 10 (27.0%) were in grades 1 and 2, 14 (37.9%) in grades 3 and 4, and 13 (35.1%) in grades 5 and 6. Age ranged from 7 to 13 years, and 19 (51.4%) were female.

### Type of products that children most frequently purchase

Overall, children wrote the names of 155 products they reported purchasing most frequently in small stores located inside and outside their schools. Products repeated by different children were included in the total. The most frequently reported purchased products were salty snacks (77, 49.7%), regular sodas (27, 17.4%), candy (18, 11.6%), pastries and cookies (10, 6.5%), fruit drinks (8, 5.2%), fruits (7, 4.5%), water (3, 2.0%), ice cream (3, 2.0%), and peanuts (2, 1.3%).

### Children’s selection of favorite product, favorite packaging, and healthiest product

Children’s responses varied when choosing their favorite product (Table 1). Of the products shown, the most popular selections were Cheetos (9, 24.4%), Fiesta Snax (7, 18.9%), Lay’s Potato Chips (6, 16.2%), and Grapette Soda (6, 16.2%). Most children, including all in grades 1 and 2, based their decision on enjoying the taste. Several added that they liked it because of a specific ingredient, *“it has chocolate,” “because of the cheese;”* or had a flavor they liked, “*I like the grape flavor.*” Children in grades 3 through 6 mentioned that the variety, large quantity, or low price of the product was the reason for their selection. For example, Fiesta Snax was liked because it included chips from different brands (i.e., Lay’s Potato Chips, Doritos, Crujitos, Max) and they perceived that the product was abundant. Premium offers (e.g., collectible, games) and characters were rarely mentioned. Several children would not choose products with fruits or vegetables on the packaging (i.e., Chupi Frut, V8 Splash), explaining that they contained a fruit or vegetable they did not like or that it had a “*sour taste*”.

*“I chose it* [Grapette] *because I like the grape flavor.”* (Girl, Grade 5–6)

“*I like this one* [Fiesta Snax] *because of the taste…and also it has chips of different flavors*.” (Boy, Grade 3–4)

*“…*[I did not choose this] *because it has kiwi and I don’t like it.”* (Boy, Grade 1–2)

The selection of favorite packaging was also varied (Table 1). The favorite packages were V8 Splash (7, 18.9%), Lay’s Potato Chips (7, 18.9%), and Grapette Soda (6, 16.2%). Children were asked to describe which packaging they like best, thus it was not surprising that they reported basing their selections on visual elements. Some explained that they liked it because of the drawings (i.e., guitar) or characters (i.e., spokes-character, licensed character). Others emphasized that the combination of colors was what caught their attention. Children in grades 5 and 6 were the only ones to mention the packaging design and style of letters (typography) when explaining their selection of favorite packaging, while those in grades 1 and 2 focused on their taste preference, rather than the appearance.

*“…because it has the characters that I see on the television.”* (Boy, Grade 1–2)

*“I like it because it looks full of life.”* (Girl, Grade 3–4)

*“…it has a combination of colors, and this little animal here.”* (Girl, Grade 5–6)

Regarding the selection of the healthiest product, most children (28, 75.7%) selected fruit drinks (contained a small percentage of concentrated fruit or vegetable). These were the only products that had fruits or vegetables on the packaging (i.e., V8 Splash, Chupi Frut). Most children believed they were made from the fruits or vegetables that appeared on the package and therefore considered them healthy and *“full of vitamins.”* Some children confused the flavor of the product with a real ingredient. For example, stating that Cheetos were made with cheese and Grapette Soda with grapes.

*“…they* [Chupi Frut and Splash] *are made from fruits, and they* [fruits] *are supposed to have vitamins.”* (Girl, Grade 5–6)

*“I chose Chupi Frut because it has strawberries, and strawberries are very healthy.”* (Girl, Grade 5–6)

### Package drawings

A total of 16 packaging elements were identified (6 visual and 10 informational) (Table 2). The most frequently drawn elements were product name made up by the child (86.5%), price (54.1%), product image (54.1%), character (43.2%), slogan (35.1%), and expiration date (35.1%). Product name, price, and product image appeared frequently in all grades. However, children in grades 1 and 2 were more aware of visual elements (Table 2). In addition to product image and character, secondary (e.g., stars, hearts) and ingredient (i.e., cheese) images were also drawn. In contrast, those in grades 5 and 6 focused more on informational elements. Slogan and the terms “New” and “Try them” were also frequently drawn. Children did not draw any exact elements from the packages displayed in the photos previously shown.

**Table 2:** Visual and informational elements included in children’s drawing of the new snack packaging*

Packaging elements	Overall	Grades 1-2	Grades 3-4	Grades 5-6
	n (%)	n (%)	n (%)	n (%)
**Visual**				
Product image	20 (54.1)	5 (50.0)	8 (57.1)	7 (53.9)
Character	16 (43.2)	3 (30.0)	10 (71.4)	3 (23.1)
Ingredient image (e.g., cheese)	11 (29.7)	3 (30.0)	1 (7.1)	7 (53.9)
Secondary image (e.g., stars and hearts)	8 (21.6)	4 (40.0)	3 (21.4)	1 (7.69)
Environment	8 (21.6)	0 (0.0)	5 (35.7)	3 (23.1)
Premium offer	5 (13.5)	1 (10.0)	2 (14.3)	2 (15.4)
**Informational**				
Product name	32 (86.5)	5 (50.0)	14 (100.0)	13 (100.0)
Price	20 (54.1)	4 (40.0)	6 (42.9)	10 (76.9)
Slogan	13 (35.1)	2 (20.0)	4 (28.6)	7 (53.9)
Expiration date	13 (35.1)	1 (10.0)	6 (42.9)	6 (46.2)
Term: “New”	11 (29.7)	2 (20.0)	2 (14.3)	7 (53.9)
Ingredient information	11 (29.7)	1 (10.0)	4 (28.6)	6 (46.2)
Manufacturer	10 (27.0)	2 (20.0)	2 (14.3)	6 (46.2)
Term: “Try them”	9 (24.3)	0 (0.0)	2 (14.3)	7 (53.9)
Nutritional information	8 (21.6)	0 (0.0)	4 (28.6)	4 (30.8)
Health claim	4 (10.8)	1 (10.0)	2 (14.3)	1 (7.69)

*Percentages were calculated based on the total number of children who included each element. One drawing could have more than one element.

## Discussion

According to our findings, salty packaged snacks are the most frequently reported food purchased by Guatemalan children and most chose based on what they thought tasted best. However, when selecting their favorite packaging and identifying the healthiest product, they relied heavily on visual elements. In their own food package drawings they most frequently included product name, followed by price, product image, and character.

In a study we conducted previously, it was found that availability and price are likely to influence children’s food choices and purchase decisions, given that the food products purchased most frequently (salty packaged snacks) are the most widely available (42% of all products) and least expensive (median price, 25th – 75th percentile; USD 0.13, 0.09 - 0.19) found in stores around public schools in Guatemala [30]. Both food availability and price have been previously linked with diet quality and obesity [31]. Low SES consumers, like the ones in our sample, tend to buy less healthy foods [32], because high-energy, low-nutrient foods are generally cheaper than low-energy, nutrient rich ones (i.e., fruits, vegetables) [33, 34].

Taste, as previously published, was the most frequently cited reason for choosing a favorite product [35–38]. Unfortunately, the most palatable foods tend to be energy-dense and include sugar, fat, and salt. Although perceived taste seemed to be most important to children when selecting their favorite product, others have found that licensed characters [15–17], unfamiliar characters [39], health claims [21], and product branding [18] can also increase taste perceptions. Accordingly, Lay’s Potato Chips and Grapette Soda were among children’s favorite products and identified as having the most liked package. Therefore, packaging can play a role in food purchasing decisions and taste perceptions.

Visual elements also appeared to be important for children when selecting their favorite packaging (i.e., character, design, colors) and the healthiest product (i.e., images). Although children rarely mentioned these elements when selecting their favorite product, they influenced their assessment of the product’s nutritional value. Similar findings have been found in Canadian elementary schoolchildren (Grades, 1 through 6), where literal interpretation of food packaging text and images often led them to mistake what foods were healthy or not [22].

Children most frequently included product names (informational element) when drawing their own package, suggesting it is a significant aspect of food packaging. Consumers associate brand names with product quality and attributes [40]. Brand names can also contribute to the reduction of anxiety by simplifying purchasing decisions [41]. Furthermore, familial food logos activate brain regions in children associated with both motivation and cognitive control [42]. In the United States and Canada, 3- to 5-year-old children have been found to prefer the taste of identical foods and drinks if the food was branded with a McDonald’s logo [18, 19]. However, the impact of brand and product names has received less attention compared to other marketing techniques included in the children’s drawings (i.e., licensed characters).

Our study has strengths and limitations. To our knowledge, this is the first published qualitative research evaluating children’s food packaging perceptions and preferences in a LMIC. However, our findings are not generalizable to all Guatemalan children because our sample was drawn from elementary public schools and therefore is representative of children of low SES. Furthermore, the group-oriented setting might have encouraged participants to respond in a socially desirable manner, although we attempted to reduce this by having a moderator skilled at working with children in this type of setting. In addition, we used activity-based focus groups and allowed the children to think about their answers and express their individual opinions through lists, picture selection, and drawings.

## Conclusion

Our study yields that Guatemalan children selected their favorite products based on taste, but research shows that food packaging can, in part, influence taste perceptions. Visual elements influenced children’s selection of their favorite packaging and healthiest products, and persuaded them to incorrectly think certain foods contained healthy ingredients like fruit. Additional research is needed to understand how less studied aspects of food packaging such as product name, price, and product image work in combination to shape children’s perceptions, taste preferences, and food choices.

As of October 2014, Guatemala lacks regulations on food marketing that protect children from being exposed to advertising that promotes high-energy, low nutrient foods. There are several marketing strategies that target children, however packaging requires special attention because it is a critical factor in the decision-making process at the POS and is often overlooked by regulatory efforts. Furthermore, several packaging elements (e.g., licensed characters, decorative designs) have been found to influence children’s taste and food preferences. Therefore, policies regulating package content and design are required to discourage consumption of unhealthy snacks. This might be another public health strategy that can aid to halt the obesity epidemic.

## Abbreviations

NCDs: Noncommunicable diseases
LMICs: Low/middle-income countries
POS: Point-of-sale
SES: Socioeconomic status
INCAP: Institute of Nutrition of Central America and Panama.
